# Genetic Diversity and SNP-Based Fingerprinting of 94 Pumpkin Cultivars: Database Establishment and Population Analysis

**DOI:** 10.3390/plants15111717

**Published:** 2026-06-02

**Authors:** Jiawei Pan, Caochuang Fang, Toheed Anwar, Kun Ma

**Affiliations:** 1 Shanghai Key Laboratory of Protected Horticultural Technology, Horticultural Research Institute, Shanghai Academy of Agricultural Sciences, Shanghai 201403, China; panjiawei199703@163.com (J.P.); biochzeu@foxmail.com (C.F.); 2 Hubei Collaborative Innovation Center for Grain Industry/Research Center of Crop Stress Resistance Technologies, Yangtze University, Jingzhou 434025, China; toheed.agric92@yahoo.com

**Keywords:** *Cucurbita* spp., whole-genome resequencing, variety identification, germplasm management, molecular breeding

## Abstract

Pumpkin (*Cucurbita* spp.) is a globally significant vegetable crop known for its high nutritional value and remarkable phenotypic diversity. Yet, the surge in new cultivar releases has overwhelmed traditional morphological descriptors, creating critical gaps in variety purity control and breeders’ rights enforcement. Despite the established utility of SNP markers as the gold standard for genetic analysis, a dedicated high-resolution molecular database for modern pumpkin cultivars remains unavailable. To address this gap, we conducted whole-genome resequencing (WGS) on 94 representative pumpkin cultivars (spanning *C. moschata*, *C. maxima*, and *C. pepo*). Clean reads were mapped to the *Cucurbita maxima* reference genome. We employed a stringent pipeline to identify genomic variants and utilized STRUCTURE software, Principal Component Analysis (PCA), and Neighbor-Joining (NJ) trees to evaluate population stratification. Linkage disequilibrium (LD) decay and DNA fingerprinting barcodes were also developed. A total of 8,873,150 high-quality variants were identified, including 7,345,007 SNPs and 1,528,143 InDels, with an average SNP density of 21,281.50 SNPs/Mb. Population analysis consistently categorized the 94 cultivars into two primary subpopulations (G1 and G2). The first two PCs accounted for 74.06% of the total genetic variance. Further analysis revealed that G1 possessed a more complex genetic architecture and slower LD decay compared to G2, suggesting distinct selection histories. Finally, we screened for highly informative biallelic SNPs to construct a DNA fingerprinting database, enabling precise sample discrimination through unique chromatic barcodes. This study fills a critical gap in pumpkin genomics by establishing a high-density SNP database and a robust fingerprinting system. These resources provide a definitive tool for variety certification, seed purity testing, and the advancement of molecular-assisted breeding in pumpkin.

## 1. Introduction

Pumpkin (*Cucurbita* spp.) ranks among the world’s most economically important and nutritionally valuable vegetable crops, distinguished by its exceptional concentrations of carotenoids, vitamins, and minerals [1,2]. The genus *Cucurbita* is characterized by high levels of phenotypic diversity and is primarily represented by five major domesticated species. It includes *Cucurbita. argyrosperma*, *Cucurbita. ficifolia*, *Cucurbita. maxima*, *Cucurbita. moschata*, and *Cucurbita. pepo* [3]. Archaeological and genetic evidence suggests that these species originated in the Americas and underwent at least six independent domestication events from distinct wild ancestors, spanning from temperate North America to tropical South America [4]. Among the domesticated taxa, *C. pepo* exhibits the most striking polymorphic diversity in fruit morphology [5], while *C. moschata* and *C. maxima* have evolved distinct environmental adaptations; the former is widely recognized for its tolerance to heat and humidity in tropical regions, whereas the latter is noted for its adaptation to cooler, temperate climates [6]. Rapid advances in global pumpkin breeding have rendered traditional morphological descriptors inadequate for ensuring the purity and authenticity of newly released cultivars. To address these challenges, molecular markers have become an essential component of modern agricultural diagnostics [7,8].

Over the past few decades, various marker systems such as Simple Sequence Repeats (SSRs) and Amplified Fragment Length Polymorphisms (AFLPs) have been widely utilized to assess genetic diversity in diverse crops [9,10]. However, single-nucleotide polymorphisms (SNPs) have recently emerged as the “gold standard” for genetic fingerprinting and population analysis [11,12]. The widespread adoption of SNPs reflects their high genomic density, biallelic nature, environmental stability, and compatibility with high-throughput automated genotyping platforms [11,13,14,15]. The evolution of genotyping technologies has led to several efficient platforms for SNP detection. Numerous SNP detection strategies, including cleaved amplified polymorphic sequence (CAPS), high-resolution melting (HRM), and kompetitive allele-specific PCR (KASP), have been established based on a variety of scientific foundations [16,17]. The practical adoption of these tools is typically determined by the balance between genotyping expenditure and overall performance [18]. Among these, the KASP assay is highly favored in crop breeding for its cost-effectiveness and high accuracy [19,20,21]. Other sophisticated methodologies, including Target SNP-seq [22] and microfluidic-based genotyping [23,24], have significantly facilitated large-scale germplasm screening.

An SNP marker-based DNA fingerprinting database is a powerful strategic tool for long-term germplasm management. It provides a scientific basis for detecting genetic redundancy in seed banks [25], ensuring the genetic integrity of commercial seed lots [26], and identifying essentially derived varieties (EDVs), which is critical for protecting plant breeders’ rights and resolving intellectual property disputes [27]. These innovations have enabled the construction of comprehensive SNP fingerprinting databases for numerous crops, such as cauliflower [28], maize [29], cigar tobacco [30], Chinese chive [31], common bean [32], and sweetpotato [33]. Within the *Cucurbitaceae* family, SNP-based molecular characterization has been successfully applied to cucumber [22], melo [34], watermelon [35], wax gourd [36], and bottle gourd [37].

The genetic diversity and evolutionary history of the genus *Cucurbita* have been extensively studied across various species and regions, providing a foundation for modern breeding programs. Research has characterized the genetic differentiation of C. *argyrosperma* in Mexico [38], traced the origins of *C. pepo* [39], and explored the metabolic profiles of feral species like *C. foetidissima* [40]. Researchers integrated morphological and SSR marker analyses of 64 *C. pepo* accessions to reveal significant phenotypic variability and distinct clustering, proving that these complementary methods are essential for accurate germplasm evaluation [41]. Within *C. moschata*, various marker systems—including PCR-SSCP, chloroplast sequences, and mitochondrial lineages—have been utilized to assess germplasm from Mesoamerica and Brazil, revealing significant variability linked to agro-morphological traits and elevational gradients [42,43,44]. While Lee et al. [45] analyzed 2071 SNPs across 610 global *C. moschata* accessions to characterize population structures and establish a 67-accession core collection, a comprehensive high-resolution molecular database specifically for modern pumpkin cultivars remains a critical gap.

Given the increasing need for variety identification and genetic resource management, this study utilizes high-density SNPs to analyze the genetic diversity and population structure of 94 representative pumpkin cultivars spanning the major groups of *C. moschata*, *C. maxima*, and *C. pepo*. Our research aimed to develop a core set of highly informative SNP markers, establish a robust DNA fingerprinting database, and evaluate the genetic diversity and population structure of these accessions. By filling a critical gap in pumpkin genomics, this work provides a definitive resource for variety certification and seed purity testing, ultimately facilitating the precision molecular breeding of this economically vital crop.

## 2. Results

### 2.1. Whole-Genome Resequencing and Mapping

The 94 representative pumpkin cultivars used for whole-genome resequencing (WGS) included 5 *C. moschata*, 58 *C. maxima*, and 31 *C. pepo* accessions (Table S1), covering the primary genetic diversity utilized in modern breeding. To ensure a unified coordinate system for variant calling, clean reads from all accessions were mapped to the *C. maxima* reference genome (HZAU version).

In total, approximately 2819 million high-quality clean reads were generated. The average mapping rate of the reads to the *Cucurbita maxima* reference genome (HZAU version HZAU GWHERBQ00000000) was 86.09%, indicating a high degree of similarity between the samples and the reference and confirming the absence of significant exogenous contamination (Table S2).

The sequencing depth across all 94 samples averaged 23.92×, with an average genome coverage of 61.64%. These results provided a robust foundation for subsequent high-accuracy variant calling and population genetic analysis (Table S3).

### 2.2. Identification and Distribution of SNPs and InDels

**Figure 1:**
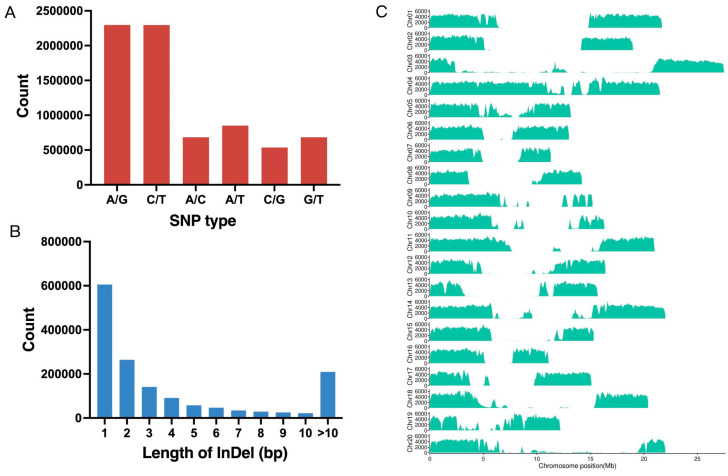
Summary and distribution of high-quality variants identified in pumpkin cultivars: (**A**) Classification and frequency of SNP types. The bars represent the counts of transitions (Ts: A/G and C/T) and transversions (Tv: A/C, A/T, C/G, and G/T). (**B**) Length distribution of InDels ranging from 1 bp to >10 bp. (**C**) Genome-wide distribution and density of SNPs and InDels across the 20 pumpkin chromosomes (Chr01–Chr20). The horizontal axis indicates the physical position along each chromosome in Megabases (Mb), and the vertical peaks represent the SNPs and InDels density within specific genomic regions.

Following a stringent filtration pipeline (GATK, depth ≥ 3, missing rate ≤ 50%, MAF ≥ 5%), a total of 8,873,150 high-quality variants were identified, comprising 7,345,007 SNPs and 1,528,143 InDels. Among the SNPs, 4,591,668 were transitions (Ts: A/G and C/T) and 2,753,339 were transversions (Tv: A/C, A/T, C/G, and G/T). The transition-to-transversion (Ts/Tv) ratio was calculated at 1.67 (Figure 1A). For the InDels, the majority (39.6%) were 1 bp in length (605,389), and the number of variants generally decreased as the insertion or deletion length increased (Figure 1B).

Across the entire genome, the mean SNP density across the whole genome was 21,281.50 SNPs/Mb. Chromosome 04 exhibited the highest SNP density (31,406.24 SNPs/Mb), while Chromosome 03 showed the lowest density (12,440.09 SNPs/Mb), suggesting non-uniform selective pressures across the genome (Table S4). The identified variants were distributed non-randomly across all 20 pumpkin chromosomes (Chr01–Chr20) (Figure 1C).

### 2.3. Development of the Pumpkin GT-seq SNP-Panel

**Figure 2:**
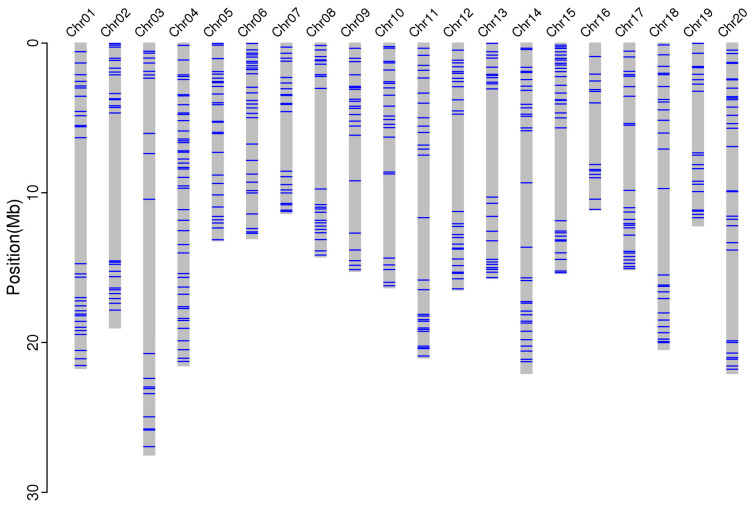
Distribution of the 500 SNPs on the twenty chromosomes of pumpkin. Blue ticks represent the locations of the SNPs.

Candidate SNPs were screened based on a minimum inter-marker distance of 50 kb and a minor allele frequency (MAF) of at least 0.2 to ensure genomic representativeness. For each selected locus, targeted amplification primers were designed using Primer3 (version 2.5.0). The design parameters were optimized with a primer length of 17–32 bp, a melting temperature (Tm) between 60 °C and 64 °C (optimal at 62 °C), and a maximum amplicon size of 500 bp, ensuring that the sequencing reads fully covered the target SNP sites. To ensure high amplification specificity, multiple primer pairs were initially designed for each locus and subsequently evaluated using e-PCR (version 2.3.12). After excluding non-specific candidates, the remaining primer pairs were further screened for potential primer-dimer formation. The final SNP panel was constructed by prioritizing primer sets with the lowest dimer-forming probability (Figure 2). The genomic distribution of these markers and comprehensive primer information are detailed in Table S5.

### 2.4. Population Structure Analysis

**Figure 3:**
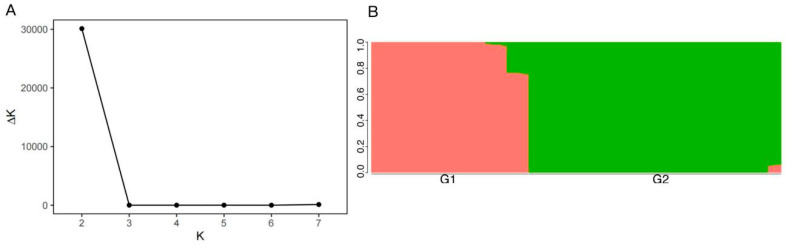
Population structure and genomic variant distribution of the 94 pumpkin cultivars: (**A**) Estimation of the optimal number of clusters (K) based on the Delta K (ΔK) method. The peak at K = 2 identifies the most likely number of subpopulations. (**B**) Integrated analysis of genetic structure and variant density. The bottom bar plot illustrates the Bayesian clustering results (K = 2) from STRUCTURE, where each vertical bar represents a cultivar, and colors indicate ancestry components (Q values) for subpopulations G1 (red) and G2 (green). The upper portion of the plot displays the genome-wide distribution of variants calculated using a 100 kb sliding window, providing high-resolution support for the differentiation between the two groups.

To evaluate the genetic relationships among the 94 pumpkin cultivars, we performed a population structure analysis using the Bayesian clustering software STRUCTURE (version 2.3.4). Based on the maximum likelihood and delta K (ΔK) values, the optimal number of clusters was determined to be K = 2 (Figure 3A). This indicates that the 94 cultivars can be primarily divided into two distinct subpopulations, designated as G1 and G2 (Figure 3B).

The ancestry components (Q values) demonstrated a clear differentiation between the two groups, though some individuals showed signals of genetic admixture. This two-group classification was further supported by combining the results with a sliding window analysis (100 kb) of variant distribution across the genome (Figure 3B).

### 2.5. Principal Component Analysis (PCA) and Phylogenetic Relationships

**Figure 4:**
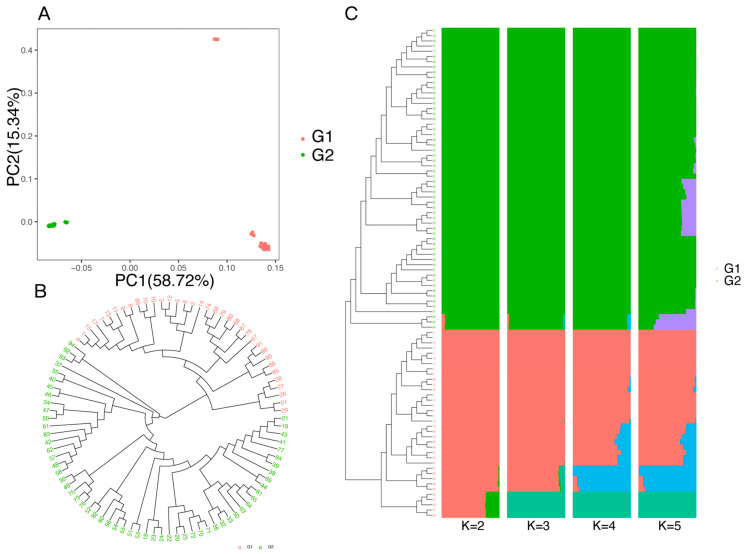
Principal Component Analysis (PCA) and Neighbor-Joining (NJ) phylogenetic tree of the 94 pumpkin cultivars: (**A**) The scatter plot displays the genetic relationships among cultivars based on the first two principal components (PC1 and PC2). Each dot represents an individual cultivar, color-coded by its assigned subpopulation: G1 (red) and G2 (green). (**B**) The tree was constructed using MEGA7 to delineate evolutionary relationships among the accessions. The cultivars are partitioned into two major clades, color-coded as G1 (red) and G2 (green). (**C**) Integrated analysis of phylogenetic relationships and population genetic structure for the 94 pumpkin cultivars. (Left) The Neighbor-Joining (NJ) phylogenetic tree illustrates the evolutionary clustering of the accessions into two primary clades. (Right) Population structure plots inferred by STRUCTURE analysis for K = 2 to 5. Each vertical bar represents an individual cultivar, where different colors indicate the estimated membership fractions (Q-values) in each inferred ancestral group.

To further validate the population stratification, we performed a Principal Component Analysis (PCA) using the PLINK 2.0 software. The PCA results were highly consistent with the STRUCTURE analysis. The first two principal components (PC1 and PC2) explained 58.72% and 15.34% of the total genetic variance, respectively. In the PCA scatter plot, the 94 cultivars were clearly clustered into two groups along the PC1 axis (Figure 4A).

Furthermore, a Neighbor-Joining (NJ) phylogenetic tree was constructed using MEGA7 to clarify the evolutionary relationships. The NJ tree branched into two major clades, which corresponded perfectly to the G1 and G2 groups identified in the STRUCTURE and PCA analyses. Within G1, the five *C. moschata* accessions (No. 26, 27, 28, 29, and 31) formed a distinct subcluster in the NJ tree, consistent with their shared morphological traits and adaptation to warm climates. This multi-method approach confirms the presence of two distinct genetic backgrounds within the tested pumpkin germplasm (Figure 4B).

To further elucidate the genetic architecture and evolutionary relationships of the 94 pumpkin cultivars, we performed an integrated analysis combining a Neighbor-Joining (NJ) phylogenetic tree with STRUCTURE clustering from K = 2 to 5 (Figure 4C). The hierarchical clustering results were highly consistent across both methods. At K = 2, the STRUCTURE analysis partitioned the entire population into two distinct genetic groups, designated as G1 (red) and G2 (green). This primary division perfectly mirrored the two major clades observed in the NJ phylogenetic tree, where the red clade corresponded to G1 and the green clade to G2. This high degree of concordance between independent analytical approaches confirms the presence of two robust and well-differentiated genetic backgrounds within the tested germplasm. As the number of inferred ancestral populations (K) increased, further genetic substratification became evident. At K = 3, a new light green ancestral component emerged specifically within the G1 subpopulation, indicating an early divergence or specific selection history within this group. This stratification in G1 was further resolved at K = 4 with the appearance of a blue genetic component, suggesting a more complex ancestral composition within G1 compared to G2 at this level. In contrast, the G2 subpopulation remained relatively uniform until K = 5, where a purple ancestral component began to appear. This sequential emergence of genetic components—first in G1 at K = 3 and K = 4 and subsequently in G2 at K = 5—indicates that the G1 subpopulation possesses a more complex internal genetic structure or a higher degree of diversification than G2. The integrated visualization demonstrates that the phylogenetic positions of the 94 cultivars are highly reflective of their multi-layered ancestral proportions, providing a robust framework for understanding the genetic diversity of the studied pumpkin accessions.

### 2.6. Linkage Disequilibrium (LD) Analysis

**Figure 5:**
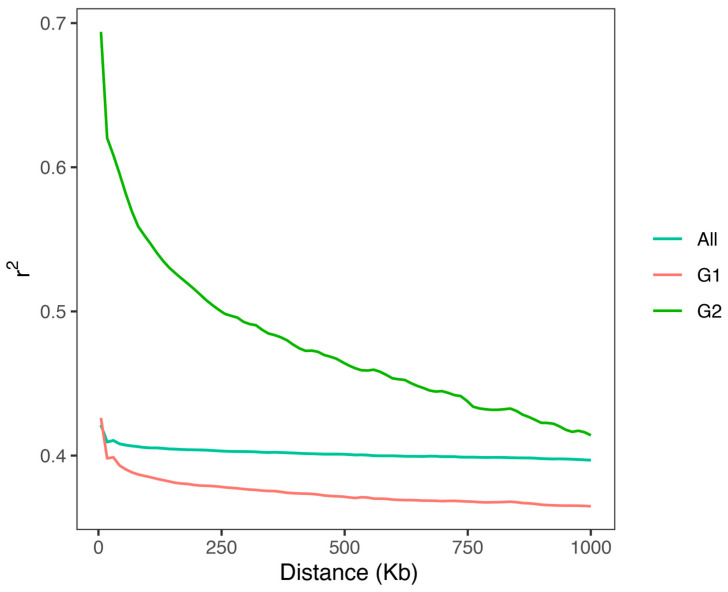
Genome-wide linkage disequilibrium (LD) decay trends in the pumpkin population. The decay of LD, measured by the squared correlation coefficient (r^2^), is plotted against the physical distance (Kb) between SNPs. The curves represent the entire population (All, teal line) and the two subpopulations, G1 (red line) and G2 (green line), calculated using PopLDdecay.

Linkage disequilibrium (LD) decay was analyzed for the entire population and for each subpopulation separately. The G2 subpopulation showed slower LD decay compared to G1, suggesting a more homogeneous genetic background and/or stronger effects of recent selection. In contrast, G1 displayed faster LD decay, indicative of higher genetic diversity and increased historical recombination. For the entire population (“All”), the r^2^ values declined sharply within the first 250 kb, reflecting relatively rapid LD decay and supporting the potential for high-resolution association mapping in pumpkin (Figure 5).

### 2.7. Establishment and Application of DNA Fingerprint

**Figure 6:**
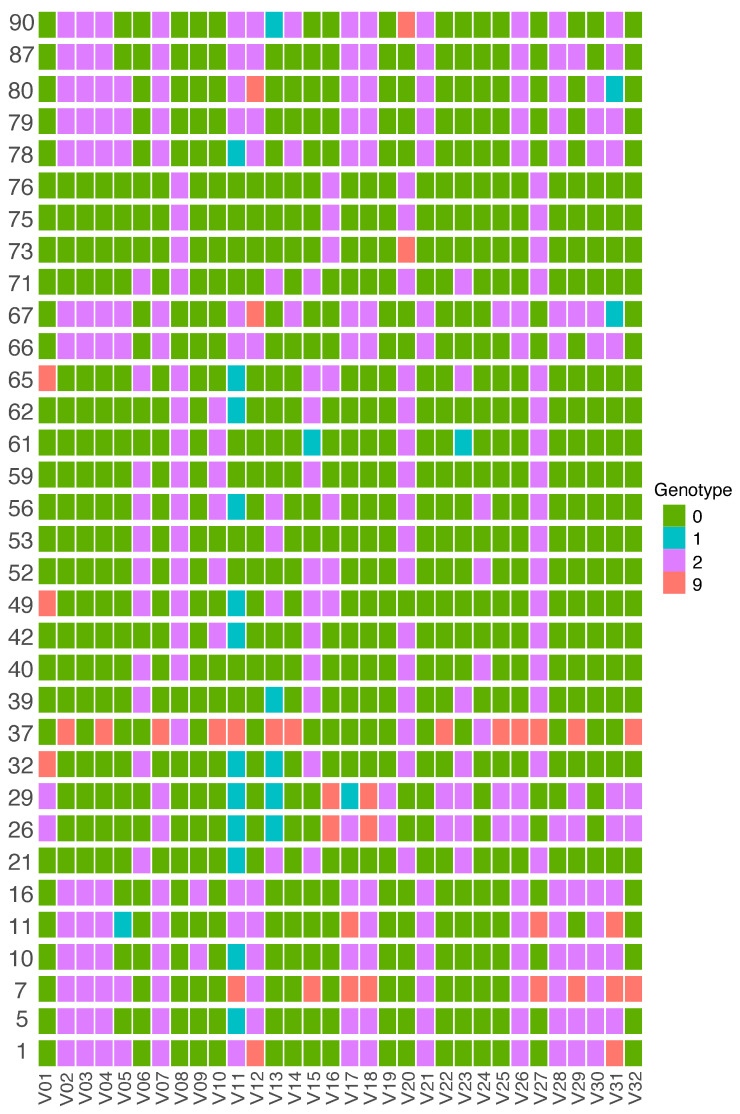
DNA fingerprinting profiles of the 33 cultivars based on 32 core SNP markers. The horizontal axis represents the 32 SNP markers, and the vertical axis represents the 33 pumpkin accessions. The pumpkin accessions and the SNPs information are given in Supplementary Tables S6 and S7, respectively. Genotypes are numerically and color-coded: “0” represents the homozygous reference genotype (0/0); “1” represents the heterozygous genotype (0/1); “2” represents the homozygous alternative genotype (1/1); and “9” represents missing data. Each row represents a unique molecular ID for a specific cultivar, highlighting the high discriminatory power of the marker set.

To establish a robust and standardized molecular identification system for the 33 pumpkin accessions, a set of 32 core SNP markers (V01–V32) was strategically selected. These markers were distributed uniformly across the genome to ensure maximum representative coverage and high discriminatory power for germplasm differentiation. Among the tested germplasm, 33 accessions representing the three major *Cucurbita* species were prioritized for DNA fingerprinting (Figure 6). These accessions, including core cultivars widely used in East Asia, were successfully assigned unique chromatic barcodes, demonstrating the system’s capacity to distinguish even closely related genotypes.

The DNA fingerprinting process involved the conversion of raw genotypic data into a standardized numerical format. Specifically, homozygous reference genotypes (0/0) were encoded as “0”, heterozygous genotypes (0/1) as “1”, and homozygous alternative genotypes (1/1) as “2”, while missing or ambiguous data were assigned a value of “9”. By concatenating the encoded alleles of the 32 SNPs in a fixed linear order, a unique digital molecular ID (fingerprint string) was generated for each cultivar. The results demonstrated that the 32-SNP panel provided sufficient resolution to distinguish all 33 cultivars (Figure 6). No two cultivars shared the same fingerprint string, indicating 100% discriminatory efficiency within the tested population. To facilitate visual comparison, these digital fingerprints were represented as a color-matrix heatmap, where each row represents a specific cultivar, and each column corresponds to a marker, highlighting the genetic polymorphisms across the collection.

For practical application in germplasm management and variety traceability, the molecular IDs were further converted into 2D Quick Response (QR) codes (Supplementary File S1). Each QR code contains essential information, including the cultivar ID and its corresponding 32-digit SNP string. This digital format allows for rapid, on-site variety identification using mobile devices.

## 3. Discussion

The rapid advancement of whole-genome resequencing (WGS) has provided an unprecedented opportunity to explore genomic variations at a high resolution, far surpassing the capacity of traditional markers such as SSRs and AFLPs [10,46]. In this study, we identified 8.87 million high-quality variants (7.34 million SNPs and 1.52 million InDels) from 94 pumpkin cultivars (Figure 1). The average SNP density of 21,281.50 SNPs/Mb is significantly higher than that reported in *C. moschata* [45], where genotyping-by-sequencing (GBS) was employed. A notable limitation of this study is the use of a single-species reference genome for read alignment across three distinct *Cucurbita* species. While high mapping rates were observed overall, divergent genomic regions—particularly in *C. pepo*, which is phylogenetically more distant from *C. maxima*—may have been underrepresented due to reference bias. This could lead to conservative variant calling and reduced sensitivity in detecting structural variation or species-specific alleles. Future studies leveraging pan-genome references will likely improve the completeness and accuracy of cross-species comparisons.

Utilizing these SNPs, a GT-seq panel of 500 high-quality, evenly distributed markers was developed (Figure 2). This panel serves as a powerful tool for breeding practices in pumpkin vegetables. Compared to traditional morphological descriptors, which often fail to distinguish modern hybrids, our SNP-based classification provides a more stable and inherited characterization. This molecular-level partitioning allows for a more objective assessment of variety purity than was previously possible. While the GT-seq SNP-panel was designed in silico based on stringent criteria, its practical performance will require empirical validation through wet-lab testing in future work.

Understanding the population structure is crucial for the effective conservation of germplasm and the advancement of molecular breeding programs [47]. An integrated analysis encompassing STRUCTURE clustering, PCA, and NJ phylogenetic trees consistently categorized the 94 pumpkin cultivars into two distinct subpopulations, designated G1 and G2 (Figure 4A,B). Interestingly, the G1 subpopulation exhibited more complex ancestral stratification at higher K values (K = 3, 4), suggesting a more diverse breeding history or a broader range of geographical origins compared to G2 (Figure 4C). The clear separation into G1 and G2 suggests a strong genetic bottleneck or distinct evolutionary paths. While G2 showed a relatively uniform genetic background, the complexity in G1 may reflect a broader exchange of germplasm or a higher degree of hybridization during the breeding process of these specific cultivars. This is analogous to the patterns observed in cauliflower, where curd characteristics and origin influenced clustering [28]. Similarly, as the K value increased from the optimal 2 to 5, the 48 tea varieties were further partitioned into more granular subgroups, revealing more intricate genetic admixture patterns and fine-scale population stratification in alignment with PCA and phylogenetic findings [48]. The 94 accessions analyzed in this study were selected from the Shanghai Academy of Agricultural Sciences (SAAS) germplasm bank to represent major cultivated types of *C. maxima*, *C. moschata,* and *C. pepo* used in regional breeding programs. However, they do not encompass the full geographic or genetic breadth of the genus. Therefore, extrapolation to broader germplasm should be done cautiously, and future efforts should integrate international collections to enhance representativeness.

Furthermore, the linkage disequilibrium (LD) analysis revealed a slower decay in G2 compared to G1 (Figure 5). The slower LD decay observed in G2 compared to G1 may reflect differences in breeding history and intensity of artificial selection. G2, largely comprising improved *C. maxima* lines, likely experienced stronger bottlenecks and selection, resulting in extended haplotype blocks. In contrast, the faster LD decay in G1 aligns with its greater species diversity and complex ancestry. As suggested by Sanjur et al. [4], the genus *Cucurbita* underwent multiple independent domestication events. The differences in LD decay between G1 and G2 may reflect distinct intensities of artificial selection during these domestication events or different levels of genetic bottlenecks encountered by these subpopulations [6]. The rapid LD decay within 250 kb observed in the overall population indicates high genetic diversity and suggests that this population is well-suited for future high-resolution association mapping of horticulturally valuable traits, such as fruit morphology and environmental adaptation [5].

As global pumpkin breeding efforts intensify, the release of numerous new cultivars has made variety identification increasingly difficult [49,50]. Traditional morphological descriptors are often insufficient to ensure variety purity due to environmental plasticity. Following the recent successful establishment of SNP-based fingerprints in tea plant [48], fiber-type hemp [51], and taro [52], we developed a robust fingerprinting database for pumpkin. By selecting highly informative core SNPs, we created unique DNA barcodes for all 33 cultivars that are widely grown and commercially significant in East Asia (Figure 6). Similar to the “chromatic schemes” used in cauliflower [28], the interpretability of our SNP-based fingerprints allows for rapid sample discrimination. This system offers a powerful tool for seed purity management, protecting plant breeders’ rights, and resolving intellectual property disputes, which are critical challenges in modern agricultural diagnostics [25,27]. The selection of highly informative biallelic SNPs ensured that even closely related cultivars within the same species could be uniquely identified. This high-resolution classification via DNA fingerprinting overcomes ‘phenotypic plasticity’, where the same variety looks different in different environments—making it a definitive tool for cultivar registration and legal protection.

The high-density SNP dataset and preliminary fingerprinting system provide a foundation for future molecular tools in germplasm management. However, operational deployment in DUS testing or seed purity control will require standardized protocols, multi-site validation, integration with phenotypic databases, and cooperation with regulatory agencies. Similarly, while rapid LD decay in the full population supports the potential for association mapping, successful implementation depends on larger, structured panels and high-quality trait data.

## 4. Materials and Methods

### 4.1. Plant Materials

A total of 94 pumpkin accessions were collected from the Shanghai Academy of Agricultural Sciences (SAAS) to represent a diverse range of germplasm (Table S1). In spring 2025, all germplasm resources were cultivated at the Zhuanghang Experimental Station, affiliated with the Shanghai Academy of Agricultural Sciences (SAAS), China. To facilitate genomic DNA isolation, immature foliage was harvested during the seedling phase.

### 4.2. DNA Extraction

Genomic DNA was extracted from fresh young leaves using the modified CTAB method [53], following the manufacturer’s protocols. The concentration and purity of the extracted DNA were evaluated using a NanoDrop™ 2000 spectrophotometer (Thermo Fisher Scientific, Waltham, MA, USA) and verified by 1.0% agarose gel electrophoresis.

### 4.3. Library Construction and Whole-Genome Resequencing

The qualified DNA samples were used to construct sequencing libraries with an insert size of approximately 350 bp. WGS was performed on the Illumina NovaSeq 6000 platform (Illumina, Inc., San Diego, CA, USA), generating 150 bp paired-end (PE) reads. The raw sequencing data were processed to remove low-quality reads, adapters, and N-containing reads to obtain high-quality clean data for downstream bioinformatics analysis.

### 4.4. Read Mapping and Variant Calling

The clean reads were aligned to the *Cucurbita maxima* reference genome (Version: HZAU GWHERBQ00000000; https://ngdc.cncb.ac.cn/gwh/Assembly/83686/show, accessed on 13 April 2026) using BWA (Burrow-Wheeler Aligner, version 0.7.15-r1140) with the MEM algorithm. Only PE reads with both ends successfully mapped were considered. The alignment results were converted to BAM format and sorted using Samtools (version 1.3.1). PCR duplicates were removed using the rmdup command in Samtools to ensure the accuracy of variant detection [54]. While this single-reference strategy facilitates consistent variant calling across accessions, it may introduce alignment bias for non-*C. maxima* samples—particularly *C. pepo*, which is phylogenetically more divergent from *C. maxima* than *C. moschata*. This could result in reduced mapping efficiency, lower coverage in divergent genomic regions, and potential under-calling of SNPs/InDels in these accessions.

Variant calling, including single-nucleotide polymorphisms (SNPs) and insertions/deletions (InDels), was performed using the Genome Analysis Toolkit (GATK, version 3.7) [55]. The HaplotypeCaller module was utilized to generate gVCF files for each sample, followed by joint genotyping using the GenotypeGVCFs module to identify potential variants across all accessions.

### 4.5. Variant Filtering and Core SNP Selection

To obtain a high-quality SNP set for fingerprinting and population analysis, the raw variants were strictly filtered. Variants were retained only if they met the following criteria: (1) sequencing depth ≥ 3 was set to minimize false-positive calls from sequencing errors; (2) genotype missing rate ≤ 50% and (3) minor allele frequency (MAF) ≥ 5% were used to select markers with high representativeness across the 94 cultivars; and (4) heterozygosity rate ≤ 60% and (5) relative heterozygosity ≤ 75% were employed to filter out potential artifacts arising from duplicated genomic regions or misaligned reads. The transition-to-transversion (Ts/Tv) ratio was calculated to monitor the quality of the SNP dataset. For the construction of the SNP fingerprinting database, a subset of highly polymorphic core SNPs was selected based on their distribution across the 20 chromosomes and their PIC (Polymorphism Information Content) values.

For genome-wide population structure analysis (STRUCTURE, PCA, NJ), high-quality biallelic SNPs were further filtered to include only those with a genotype missing rate ≤ 10%, minor allele frequency (MAF) ≥ 5%, and without significant deviation from Hardy–Weinberg equilibrium (*p* > 1e^−6^).

Heterozygosity filters (observed heterozygosity ≤ 60% and relative heterozygosity ≤ 75%) were applied to exclude putative paralogous loci and regions prone to misalignment, which are known to inflate heterozygosity estimates and bias population inference. These thresholds were determined empirically based on the distribution of heterozygosity across the genome.

### 4.6. Development of the SNP-Panel

For the SNP-panel development, a total of 3748 candidate SNPs were first prioritized based on an inter-marker distance exceeding 50 kb and a minor allele frequency (MAF) of at least 0.2. Targeted primers for these loci were designed using Primer3 (version 2.5.0) with the following constraints: a primer length of 17–32 bp, a melting temperature (Tm) between 60 °C and 64 °C (optimal at 62 °C), and a maximum amplicon size of 500 bp, ensuring that sequencing reads effectively spanned the target sites [56]. Initially, three candidate primer pairs were generated per SNP, and their amplification specificity was rigorously assessed using e-PCR (version 2.3.12). After excluding non-specific primers, the remaining 8559 pairs were further screened for potential primer-dimer formation. Ultimately, 500 primer pairs with the lowest dimer-forming probability were selected to constitute the final SNP panel. The comprehensive details regarding the SNP loci and primer sequences are provided in Table S5.

### 4.7. Population Structure and Principal Component Analysis

The population genetic structure was analyzed using a Bayesian model-based clustering method in STRUCTURE (version 2.3.4) [57]. The number of subpopulations (K) was varied from 1 to 10, and the optimal K value was determined based on the maximum ΔK using the Evanno method [58].

Principal Component Analysis (PCA) was performed using PLINK (version v1.90p) [59] to further evaluate the genetic relationships and clustering patterns of the pumpkin accessions. The PCA plot was generated based on the first and second principal components (PC1 and PC2) to visualize the genetic distance between individuals.

### 4.8. Phylogenetic and Linkage Disequilibrium Analysis

A phylogenetic tree was constructed using the Neighbor-Joining (NJ) method in MEGA7 (version 7.0) [60] based on the calculated genetic distance matrix. The resulting tree was visualized and annotated using the R package ggtree (version 1.7.10) [61]. Linkage disequilibrium (LD) decay was analyzed using PopLDdecay (version 3.41) [62]. The squared correlation coefficient (r^2^) between pairs of SNPs was calculated, and the LD decay curve was plotted against the physical distance (up to 1000 Kb) to assess the rate of LD decay across the pumpkin genome and within different subpopulations.

### 4.9. Construction of DNA Fingerprints and Generation of 2D Barcodes

A total of 32 core SNP markers were strategically selected to construct DNA fingerprints for 33 pumpkin cultivars. These markers were chosen based on their uniform physical distribution across the genome to ensure high discriminatory power and representative coverage of genetic diversity. Candidates were prioritized based on high-quality flanking sequences for optimal primer design, with melting temperatures (Tm) ranging from 60 °C to 64 °C, high specificity, and a low probability of primer-dimer formation. This ensures the reliability and reproducibility of the markers across different genotyping platforms.

Raw genotypic data were standardized into a numerical format: homozygous reference genotypes (0/0) were encoded as “0”, heterozygous genotypes (0/1) as “1”, homozygous alternative genotypes (1/1) as “2”, and missing or ambiguous data were represented by “9”. For each cultivar, these 32 encoded SNP values were concatenated in a fixed linear order to generate a unique digital molecular identity (ID), providing a concise summary of the whole-genome genetic profile. To enhance practical utility and variety traceability, the resulting fingerprints were visualized as color-matrix profiles and further converted into 2D Quick Response (QR) codes using the online platform Caoliaoerweima (http://cli.im/). Scanning these barcodes allows for rapid access to the cultivar name and its corresponding molecular fingerprinting data.

## 5. Conclusions

This study demonstrates that whole-genome resequencing is a powerful tool for analyzing the genetic diversity of pumpkin (*Cucurbita* spp.). By resequencing 94 cultivars, we identified 8.87 million variants, enriching the genomic data available for modern pumpkin germplasm. Population analysis categorized the accessions into two distinct subpopulations (G1 and G2) with contrasting selection histories. We established a proof-of-concept DNA fingerprinting system using 32 core SNPs and unique QR barcodes, achieving 100% discriminatory efficiency. These resources provide a standardized molecular tool for variety certification and seed purity testing. Ultimately, this work establishes a solid foundation for germplasm management and the advancement of marker-assisted breeding in pumpkin.

## Data Availability

Data are contained within the article and Supplementary Materials.

## Supplementary Materials

The following supporting information can be downloaded at: https://www.mdpi.com/article/10.3390/plants15111717/s1, Table S1. Species distribution and source of the 94 pumpkin accessions. Table S2. Summary of sequencing data and mapping rates. Table S3. Summary of genomic coverage and coverage depth. Table S4. Summary of variation quantity and density. Table S5. Primer sequences for the GT-seq panel of the 500 SNPs. Table S6. List of the 33 pumpkin accessions used for fingerprinting. Table S7. DNA fingerprinting profiles of 33 pumpkin accessions derived from 32 core SNPs. Supplementary File S1. 2D Quick Response (QR) codes’ fingerprint profiles.
